# Intraoperative ultrasound to aid resection of a peritorcular meningioma: a technical note

**DOI:** 10.1093/jscr/rjab462

**Published:** 2021-10-31

**Authors:** Kapil Mohan Rajwani, Jose Pedro Lavrador, Anna Oviedova, Richard Gullan, Keyoumars Ashkan, Francesco Vergani, Ranjeev Bhangoo

**Affiliations:** Department of Neurosurgery, King’s College Hospital, London, UK; Department of Neurosurgery, King’s College Hospital, London, UK; Department of Neurosurgery, King’s College Hospital, London, UK; Department of Neurosurgery, King’s College Hospital, London, UK; Department of Neurosurgery, King’s College Hospital, London, UK; Department of Neurosurgery, King’s College Hospital, London, UK; Department of Neurosurgery, King’s College Hospital, London, UK

## Abstract

Surgery for meningiomas involving dural venous sinuses is challenging. We describe a case of a peritorcular meningioma involving major venous sinuses, which was removed using a venous sparing approach with the aid of intraoperative ultrasound. We found ultrasound to be a useful adjunct as it enabled us to get real-time information about the location of venous structures, their function and demonstrate dynamic changes in blood flow during surgery.

## INTRODUCTION

Peritorcular meningiomas are rare entities; they represent ~1% of intracranial meningiomas and, because they often involve multiple branches of major venous sinus, they are considered to be particularly challenging [1–3]. They arise from, invade or are attached to a wall of the torcular herophili, the site of confluence of the superior sagittal, occipital, straight and both transverse sinuses [3]. By compressing or invading dural venous sinuses, peritorcular meningiomas compromise venous flow at the torcular. However, as they are relatively slow growing, this usually allows venous collaterals to form [4].

Interruptions to venous drainage of the brain can result in venous congestion, cerebral oedema, infarction and intraparenchymal haemorrhage [1, 4]. Therefore, preservation of the venous system is important to avoid serious complications, and a thorough knowledge of the venous functional anatomy is essential to execute a safe and effective operative strategy. Real-time intraoperative imaging can provide useful information in planning the management of cerebral venous structures as they are highly dynamic in nature [5]. Intraoperative ultrasound (ioUS) is truly real-time, permits operating under direct guidance and is relatively cheap when compared with other intraoperative imaging modalities like computed tomography (CT) or magnetic resonance imaging (MRI) [6]. We describe a case of a torcular meningioma involving major venous sinuses, which was removed using a venous sparing approach with the aid of ioUS.

## CASE REPORT

**Figure 1:**
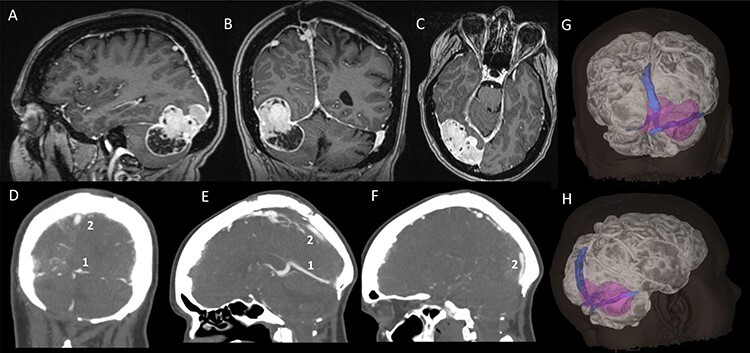
Pre-operative imaging; (**A**–**C**) sagittal, coronal and axial T1-weighted MRI images, with gadolinium demonstrating a supra-infratentorial peritorcular tumour; (**D**–**F**) CT venogram showing the patency of the straight sinus (number 1) and the superior sagittal sinus (number 2); (**G** and **H**) 3D model of the tumour, brain and dural venous sinuses generated using both the MRI images and CT venogram.

A 48-year-old, right-hand-dominant lady was under surveillance for a known right tentorial meningioma. At the age of 19, she had a posterior fossa craniectomy and resection of medulloblastoma, which was followed by cranio-spinal radiotherapy. She developed intracranial meningiomas presumably as a consequence of radiation exposure. The tentorial meningioma had progressed radiologically on surveillance imaging over 5 years. It had grown around the right transverse sinus and involved the torcula, sagittal sinus and, to some extent, the straight sinus. Cerebral CT venography showed the meningioma was causing stenosis of the posterior third of the superior sagittal sinus (Sindou Type III sinus invasion) and the right transverse sinus was almost completely occluded (Type IV). Filling defects were seen in the torcula, however, the left transverse sinus and straight sinus were predominantly patent (Fig. 1). After discussion of her case at our neuro-oncology multi-disciplinary meeting, we offered her surgical resection of the meningioma.

With the patient under general anaesthetic, she was positioned prone with her head in Mayfield pins. Through a hockey-stick incision incorporating the previous midline posterior fossa scar, a neuro-navigation guided right occipital craniotomy was fashioned. She previously had a posterior fossa midline craniectomy. The posterior fossa cranial defect was identified and was extended to fashion a right suboccipital craniectomy, leaving a strip of bone over the right transverse sinus. Two-dimensional ioUS images (B-mode) were used to identify the tumour, dural folds and vessels prior to dural opening, and we tailored the durotomy accordingly. Tumour infiltrating the dura was seen both above and below the tentorium, and this was resected.

**Figure 2:**
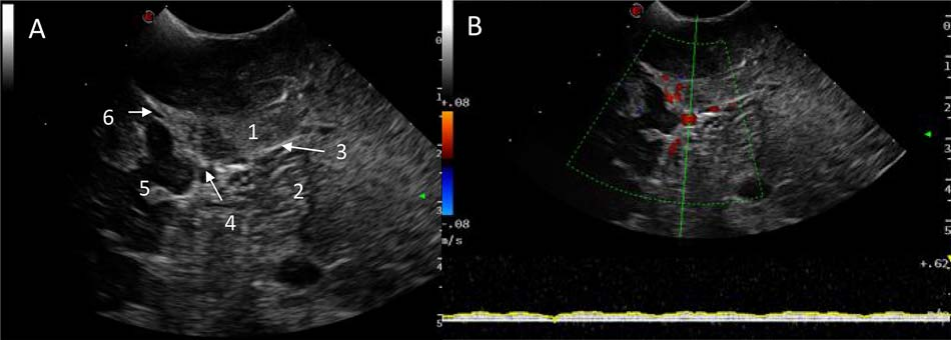
IoUS initial assessment; (**A**) B-mode image of the surgical field; 1, supratentorial tumour; 2, infratentorial tumour; 3, tentorium; 4, straight sinus; 5, contralateral occipital lobe; 6, falx; (**B**) triplex Doppler of the straight sinus: combined 2D image with overlay of the colour and pulse wave Doppler demonstrating unidirectional flow from the vein of Galen and the tributary veins of the tentorium.

Ultrasound (B-mode) was used to identify the tumour margins, tentorium and venous structures (Fig. 2). Combined with the B-mode, Doppler ultrasound (US) was employed to assess the presence and direction of flow in the right transverse sinus, torcula and superior sagittal sinus. There was presence of blood flow in all these dural venous sinuses, however, the flow within the straight sinus was only unidirectional towards the torcula from the vein of Galen and from the tentorium tributary veins. The unique feature of the intracranial venous sinuses is that blood flow is bi-directional as they lack valves. Hence, this finding suggests there was a degree of venous insufficiency due to obstruction of the venous sinuses engulfed by the meningioma (Fig. 2).

All this information was taken into account as we planned our operative strategy. The use of US to visualize the tumour borders enabled us to efficiently and safely debulk the meningioma centrally using bipolar diathermy, suction and ultrasonic aspirator. This allowed the capsule to collapse down in both the supra- and infra-tentorial compartments, and we identified a satisfactory tumour/brain cleavage plane without excessive cerebral and cerebellar manipulation. Large parts of the tumour were removed piecemeal, all the while using multimodal ioUS to identify and meticulously preserve the venous structures.

**Figure 3:**
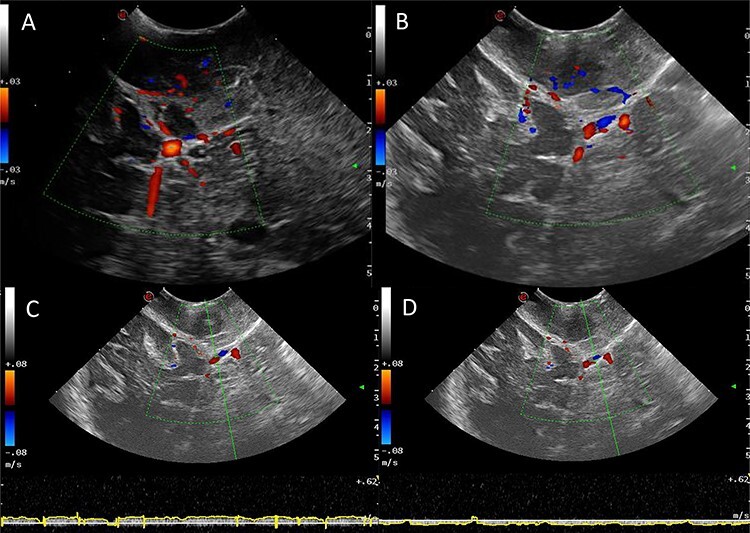
Intraoperative images at the end of resection; Duplex US images (2D image with overlay of colour Doppler) of the initial appearances of the straight sinus **(A)** and appearance at the end of resection **(B)**; a bidirectional flow in the straight sinus is apparent after tumour resection, this was not present prior to tumour debulking; the Triplex Doppler confirms the bidirectional flow in the straight sinus **(C** and **D)**.

**Figure 4:**
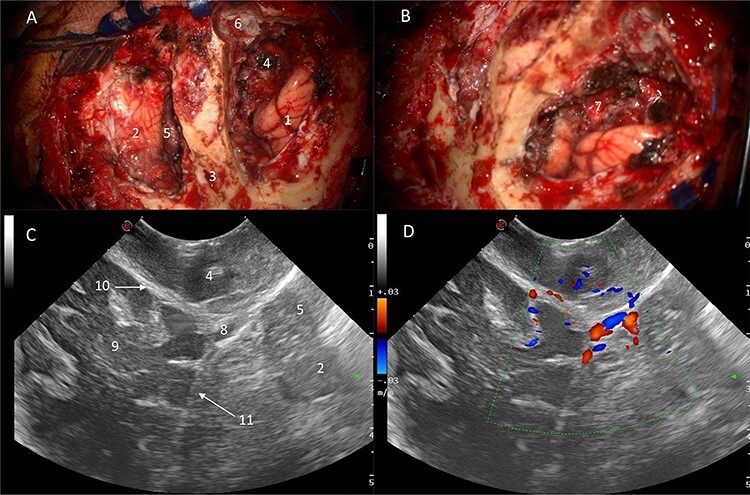
Correlation of US appearance and gross anatomy; (**A** and **B**) intraoperative picture of the surgical field at the end of resection; (**C** and **D**) final US assessment—B-mode (C) and duplex (D); 1, ipsilateral occipital lobe; 2, ipsilateral cerebellar hemisphere; 3, strip of bone on top of the transverse sinus; 4, supratentorial resection cavity; 5, infratentorial resection cavity; 6, torcular; 7, supra-infratentorial communication of the tumour via the tentorium; 8, straight sinus; 9, contralateral occipital lobe; 10, falx cerebri; 11, tentorium.

Power and colour Doppler were used throughout to obtain real-time information about the location and function of dural venous sinuses. Blood flow was preserved in the sinuses and, as we debulked the tumour, we were able to demonstrate bi-directional flow within the sinuses (Fig. 3). This suggested resolution of venous obstruction and indicated the dural venous sinuses had regained their functionality. Medially tumour was found invading the torcula and right transverse sinus, and a decision was made to leave the residual tumour and to preserve the sinuses (Fig. 4). We achieved a Simpson Grade 4 resection without any intraoperative complications.

Histopathology confirmed a World Health Organization Grade 1 meningioma. The patient made an uneventful recovery, and post-operative imaging showed a small tumour residual attached to the dural sinus.

## DISCUSSION

Numerous studies have investigated the utility of ioUS imaging for neoplastic lesions in neurosurgery [7–10]. There are two main reasons why we found ioUS to be a useful adjunct in the management of venous structures in this case. Firstly, there is variability in the exact location of dural venous sinuses among patients and they potentially extend several centimetres from the midline [5]. Hence, there is a risk of inadvertently damaging these venous structures. US enabled us to identify the tumour margins, dural folds and vessels even prior to dural opening and to tailor the durotomy accordingly. We were then able to use the real-time information about the location of vessels and sinuses to guide our resection.

Secondly, cerebral veins are also highly dynamic in nature, and they respond to several factors, including head positioning, intracranial pressure, bone flap removal and mechanical stimuli (e.g. tumour compression) [5]. Doppler US was useful in obtaining information about changes in venous blood flow and the direction of flow. Prior to tumour debulking, the flow within the dural venous sinuses engulfed by the meningioma was unidirectional, suggesting there was a degree of venous insufficiency. Towards the end of the procedure, we were able to demonstrated bi-directional flow within the sinuses, which suggests they had regained their functionality.

Multimodal ioUS enables quick, real-time, cost-effective intraoperative imaging. This report presents the usefulness of ioUS for resection of a torcular meningioma by using a venous sparing approach. We were also able to demonstrate improved blood flow in the dural venous sinuses at the end of the procedure. We found ioUS to be a useful adjunct and recommend its use in the resection of meningiomas involving major venous sinuses.

## CONFLICT OF INTEREST STATEMENT

None declared.

## FUNDING

None.
